# Temporary Hydrostatic Splint Therapy and Its Effects on Occlusal Forces

**DOI:** 10.3390/medicina60071051

**Published:** 2024-06-26

**Authors:** Mante Kireilyte, Povilas Ancevicius, Ausra Baltrusaityte, Vita Maciulskiene, Gediminas Zekonis

**Affiliations:** 1 Department of Prosthodontics, Faculty of Dentistry, Academy of Medicine, Lithuanian University of Health Sciences, LT-50106 Kaunas, Lithuania; mante.kireilyte@lsmu.lt (M.K.);; 2 Dental Centre of Siauliai, LT-76332 Siauliai, Lithuania; 3 Department of Dental and Oral Pathology, Faculty of Dentistry, Academy of Medicine, Lithuanian University of Health Sciences, LT-50009 Kaunas, Lithuania

**Keywords:** occlusal splints, dental occlusion, balanced dental occlusion, temporomandibular joint disorders, bite force

## Abstract

*Background and objectives:* This study analyzed and compared the distribution patterns of occlusal forces using T-Scan III before and after the hydrostatic temporary oral splint (Aqualizer Ultra) therapy in healthy subjects and subjects with temporomandibular disorders (TMDs). *Materials and Methods*: Fifty-one subjects were divided into groups based on anamnesis and responses to the Fonseca questionnaire. The first group, non-TMDs group (n = 19), and the second group, TMDs group (n = 32), had mild-to-severe TMDs, as identified by the Fonseca questionnaire. The non-TMDs group had an average age of 25.4 years (SD = 4.8, range 20–38) with 15 females (78.95%) and 4 males (21.05%). The TMDs group had an average age of 27.4 years (SD = 7.0, range 22–53) with 25 females (78.125%) and 7 males (21.875%). T-Scan III device was used for occlusal analysis before and after hydrostatic splint usage. *Results:* Significant differences were observed in the TMDs group for anterior and posterior right percentages of forces before and after hydrostatic splint usage. The analysis of force distribution per sector before and after hydrostatic splint therapy showed no significant differences in the non-TMDs group. Analysis of force distributions in the entire study population before and after hydrostatic splint therapy showed significant differences in the anterior and posterior right regions. Occlusal force increased by 32–56% in the front region and decreased in the posterior area after hydrostatic splint usage. *Conclusions*: Hydrostatic splint therapy is recommended as a part of full-mouth rehabilitation treatment for all patients regardless of the severity of TMDs.

## 1. Introduction

Since the late 19th century, various occlusal concepts and philosophies for full-mouth rehabilitation have been invented in relation to achieving a supposedly optimal occlusion [1]. There had been decades of controversy about what centric relation actually means and how to determine it [1], but dental occlusion is still a topic open for discussion in dentistry.

The first documented study on bite force in the 17th century is attributed to the work of Italian scientist Giovanni Alfonso Borelli. Borelli, often considered the father of biomechanics, conducted extensive studies on the mechanics of animal movements and the forces involved. In 1987, with the start of the modern biotechnology era, Maness et al. developed a high-precision, easy-to-use computerized system called T-Scan, which can analyze forces of occlusal contacts in real time [2]. This invention provided researchers with an objective tool to quantitatively and qualitatively analyze occlusal contact forces [3,4,5], instead of the use of conventional, non-digital indicators [4]. In 1991, Moini and Neff [6] were the first researchers to use this computerized device to clinically examine the accuracy and reproducibility of the occlusal contacts in ten asymptomatic subjects. The results showed the maximum accuracy and reproducibility for all contacts in all subjects. In 1997, Cartagena et al. [7] proposed that the maximum load was located on the second lower molars, decreasing in posterior–anterior direction, and that the average occlusal load was greater on the left side. Later, Ferrato et al. [8,9] showed that the maximum load was located in the area of the first upper molars at the mesial–palatal cusp. In 2013, Ma et al. [10] partly confirmed the Ferrato group’s data: the regions from the first premolar to the second molar teeth were the occlusal force centers at the intercuspal position, and the second molar on the left and right sides took the largest part of the percentage force. However, if carried out correctly, the digitized occlusion examination is simple, repeatable, and largely operator independent, which provides a huge advantage in using this digital system for both clinical and research applications [3].

As a consequence of dramatically increasing the prevalence of TMDs from fourteen (per 10,000) to twenty-six (per 10,000) over the past ten-year period [11], the treatment of TMDs has become indispensable in today’s dental routine. According to the most recent literature, the global incidence of TMDs is 34%. Signs and symptoms of TMDs are most common around the age of 30–35 years for individuals primarily complaining of temporomandibular joint disk displacements with or without pain, and around the age of 50–55 years for those with degenerative joint disorders. Additionally, the prevalence of TMDs is higher in females compared to males, with the female-to-male ratio ranging from 1.09 in Europe to 1.56 in South America, indicating that the female group is, on average, 9% to 56% larger than the male group in various regions [12]. Other studies, such as the 2023 review by Alrizqi and Aleissa, have similar findings, highlighting the higher prevalence of TMDs among females and the significant impact of psychological factors and age on TMD [13]. These insights were further supported by the subsequent meta-analysis by Zielinski et al. (2024), which also emphasized the demographic variations and gender disparities in TMD prevalence [12].

Treatment of TMDs can be surgical or conservative. The conservative therapy includes either soft or hard splints for the upper/lower jaw [14]. Despite the fact that some studies have shown hard splints to be more efficient for TMDs [15], soft splint therapy (e.g., hydrostatic splint) results in earlier improvement of some TMDs symptoms [16]. The aim of such splints is to eliminate all tooth-to-tooth contacts. Once the proprioceptive guidance is neutralized as the dominant factor in functional mandibular placement, the masticatory muscles are not forced into position by the way the teeth fit together. The hydrostatic splint fluid system re-equilibrates and bilaterally balances the mandible by putting the condyles in a more central position in the mandibular fossa in order to improve the neuromuscular balance and reduce muscle activity to relieve the TMDs [14,17].

The research hypothesis of this study is that the use of the hydrostatic splint will improve the distribution of occlusal forces in both non-TMD and TMD groups, leading to a more balanced occlusion. The anti-hypothesis is that the hydrostatic splint will not have a significant effect on the distribution of occlusal forces, and the occlusal imbalance will remain unchanged in both groups.

The purpose of this study was to establish the significance of hydrostatic splint usage prior to full-mouth rehabilitation for any individual. The study involved objective analysis and comparison of occlusal force distribution patterns using T-Scan III before and after the hydrostatic splint therapy on healthy subjects and on subjects with TMDs.

## 2. Materials and Methods

### 2.1. Ethical Aspects

This trial was conducted from December 2022 to January 2023 in the dental clinic MK Dental Studio in Marijampole, Lithuania. The participants signed an informed consent form approved by Kaunas Regional Biomedical Research Ethics Committee (No. BE-2-48). All procedures were fully explained to the participants, who provided written informed consent. All procedures followed the standards of the Helsinki Declaration of 1964, as revised in 2013.

Study registered at ClinicalTrials.gov (NCT05827263).

### 2.2. Study Sample

In this clinical study, fifty-one dentate adults were recruited from the regular patients of MK Dental Studio. To standardize the procedure, the participants were screened by a single trained dentist during the selection process. The study population included healthy subjects with Angle Class I and a normal line of occlusion without malpositioned or rotated teeth. Additional inclusion criteria included the following: complete permanent dentition except for the third molars; no fixed prosthesis; no dental caries; no restorations on the occlusal surfaces of molars and premolars extending more than one-third of the surface; no restorations on incisal edge; no tenderness on percussion of any teeth; and no history of previous endodontic and orthodontic treatment, extensive maxillofacial surgery, or systematic neurological disorders. Exclusion criteria were the presence of orofacial pain that limits mouth opening, malocclusion (e.g., open bite, increased overjet or reverse overjet, cross bite), and skeletal anomalies with occlusal disturbance.

The participants completed a self-reported questionnaire containing information about personal, medical, and dental histories. In addition, the patients completed the “bruxism” scale (BRUX) and Fonseca questionnaires. The BRUX scale was derived from the following items: clenching at night, grinding at night, clenching in daytime, and grinding in daytime. Response options, using an ordinal 5-point Likert-type scale, included the following: 0 = never, 1 = sometimes, 2 = regularly, 3 = often, and 4 = always. An average score (range: 0 to 4) of the 4 items was computed [18,19]. Fonseca at al. [20] developed the second questionnaire to identify potential participants having TMDs. Participants were instructed to reply to ten questions about difficulty during mouth opening and lateral deviations, pain during mastication, headache, neck and temporomandibular joint (TMJ) pain, TMJ sounds, parafunctional habits, malocclusion perception, and perception of stress. They had to choose one of the following answers indicating different degrees of TMDs: yes (ten points), no (zero points), and sometimes (five points). The sum of the points was used to classify the participants into four categories: non-TMDs (0 to 15 points), mild TMDs (20 to 40), moderate TMDs (45 to 60), and severe TMDs (70 to 100) [20,21]. On the basis of the TMDs diagnosis, participants were divided into two groups: non-TMDs group and TMDs group (patients diagnosed with mild, moderate, or severe TMDs).

### 2.3. T-Scan Occlusal Recording

Occlusal information was obtained using the T-Scan device (T-Scan III, Tekscan Inc., Norwood, MA, USA; software version 7.01T). T-Scan computerized occlusal scans of the participants were taken by the first author (K.M.) under the guidance of the coauthor (A.P.). Participants were seated upright in a comfortable position and asked to relax [3,18]. They were instructed to practice mandibular opening and closing movements at least three times before recording the procedure [3,22,23]. A new appropriately sized T-Scan HD sensor was used for each patient [3]. Mesiodistal width of the right maxillary central incisor was measured with a digital caliper (Tovarna meril Kovine d.d.) to customize the dental arch dimension [3,23]; this was followed by intra-oral sensitivity of the sensor calibration in the software with a maximum of two to three red dots (high-pressure areas) for the trial bite [3,22,23]. Each participant was asked to bite into the intercuspal position using maximum force with a 100 µm thick sensor foil placed intraorally [3,22,23,24]. Occlusal registration was repeated three times to confirm the findings [24].

### 2.4. Using the Hydrostatic Splint

A hydrostatic splint is a preconfigured device with two distilled water-filled chambers positioned in the posterior occlusion connected over a tube in the front. The device works through the principle of Pascal’s law, which states that a pressure change at any point in a confined incompressible fluid is transmitted throughout the fluid, such that the same change occurs everywhere [14,25]. Generally, within the first few minutes, the mandible shifts to the position most comfortable for the muscles to function and a neuromuscular balance develops [26].

In the present study, the hydrostatic appliance, Aqualizer Ultra, medium volume (two mm thick) was chosen because low volume (one mm thick) is designed for individuals who experience limited jaw opening and is also an excellent option for obtaining precise muscle-focused bite registrations for splints; in contrast, high volume (three mm thick) is employed in situations where a patient presents with an excessive amount of freeway space, or when an increased vertical dimension is necessary to bridge the gap between the upper and lower occlusal surfaces. In the majority of instances, the hydrostatic splint with medium volume is the recommended choice, covering around 90% of cases [26]. The usage of the hydrostatic splint was implemented immediately after the first occlusal recordings with the T-Scan were obtained. The hydrostatic splint was removed from the package and placed symmetrically between the upper lip and the oral vestibule of the maxilla for the most comfortable position. Patients were instructed to keep the fluid pads between the posterior teeth. Patients were not allowed to clench and were asked to be aware of any change in sensation anywhere in the head, neck, shoulders, and upper back. Patients’ symptoms (muscle pain in the jaw, head, neck, shoulders, headache) were monitored every ten minutes. These symptoms were noted because changes in occlusion can affect the entire neuromuscular system, including the head, neck, shoulders, and upper back. The awareness of these changes helps in identifying any immediate neuromuscular adaptations or discomfort caused by the splint, which is crucial for understanding its overall impact. After thirty minutes, the hydrostatic appliance was removed, and patients were asked to keep their mouth open until the T-Scan was placed in the right position. Occlusal registration was repeated accordingly three times. The occlusal data of patients were transferred into a spreadsheet for further analysis.

### 2.5. Occlusion Analysis

Measured occlusal data included force distribution, contributing teeth at maximum intercuspation, maximum percentage force on teeth, and center of force location for arch symmetry. T-Scan patterns analyzed the arch’s occlusal force at maximum intercuspation, grouped into three regions: anterior (canine to canine, numbers 13–23), posterior right (first right premolar to third right molar, numbers 14–18), and posterior left (first left premolar to third left molar, numbers 24–28).

No changes to methods and to trial outcomes were made after the trial commencement.

### 2.6. Statistical Analysis

Statistical analysis utilized IBM SPSS Statistics v27.0 (IBM Corp., Chicago, IL, USA). The Wilcoxon signed-rank test assessed significant differences (*p* < 0.05) before and after hydrostatic splint use. Descriptive statistics presented patients’ characteristics. Due to non-normal distribution (Kolmogorov–Smirnov test), values were expressed as median [IQR, 25th–75th percentile]. Categorical variables were summarized using counts and percentages. The median [IQR] was calculated for occlusal force percentages for single dental elements, sections, and sides. The number of contributing teeth and maximum percentage force on teeth were determined for participants.

## 3. Results

The sample size was determined using an alpha level (α) of 0.05 to ensure statistical significance. Calculations were performed with IBM SPSS Statistics v27.0, utilizing the dependent sample *t*-test (paired *t*-test), which is appropriate for comparing paired data. We selected an effect size of 0.5, which is considered a medium effect size in clinical studies, to detect meaningful differences. This effect size is widely accepted as appropriate for detecting clinically relevant changes. The results demonstrated statistically significant differences in force distributions before and after hydrostatic splint therapy in the TMDs group, with statistical power exceeding 0.8, indicating a high likelihood of correctly rejecting the null hypothesis. Specifically, for the entire population, the statistical power was 0.83 for detecting significant differences in the anterior region before and after using the splint, confirming the adequacy of our sample size for detecting the expected effects.

The participants’ demographic data are described in Table 1.

### 3.1. BRUX and Fonseca Questionnaires

The percentage distribution of all participants’ responses to the four questions of the BRUX scale are shown in Table 2. The mean total BRUX score was 1.24, SD = 0.8. According to the Mann–Whitney Test, there were no statistically significant differences (*p* = 0.799) in bruxism activities in the non-TMDs group compared with the TMDs group. The BRUX questionnaire Reliability Statistics, Cronbach’s Alpha 0.73, indicate acceptable reliability. In the non-TMDs group, the median score on the BRUX scale was 1, which means that patients sometimes grind/clench their teeth while awake/during sleep. In the TMDs group, the median score on the BRUX scale was 1.5, which means that patients more often sometimes grind/clench their teeth while awake/during sleep.

According to Fonseca’s anamnestic index, 62.7% of all the participants demonstrated mild-to-severe TMDs symptoms (see Table 3). The median of the collective score of the questionnaire in the non-TMDs group was 15 [10.0–15.0], which did not indicate any TMDs. The median in the TMDs group was 32.5 [20.0–50.0], which indicated mild TMDs. The analysis of the questionnaire showed a statistically significant difference in the groups of the study (*p* < 0.001).

### 3.2. Occlusal Forces for Single Dental Elements

Force values expressed as a percentage for individual teeth were considered for this assessment (see Table 4). The third molars were not included in the evaluation.

In the non-TMDs group, the greatest occlusal loading was detected in the area of the left first upper molar with a median percentage of 11.7% strength. On the other elements, proceeding in the posterior–anterior direction (second and first premolars, canines, lateral incisors), progressively inferior values were registered up until the central incisors. Central incisors had similar occlusal loadings to premolars.

There were no particular differences in the occlusal loads detected in the TMDs group before the hydrostatic splint. The greatest occlusal loadings were also detected in the molar area; for the other elements, proceeding in the posterior–anterior direction, progressively inferior values were registered up until the central incisors.

After using the hydrostatic splint, the occlusal loads were reduced only by 0.3–0.4% in three areas (tooth Nos. 16, 13, and 24) in the non-TMDs group. For the other elements, superior values were registered. In the TMDs group, particular differences can be detected in second premolars—second molar areas—where all occlusal loads were reduced.

Statistical analysis using the Wilcoxon signed-rank test showed no significant difference in the total occlusal force distribution before and after using the hydrostatic splint in both non-TMD and TMD groups.

**Figure 1:**
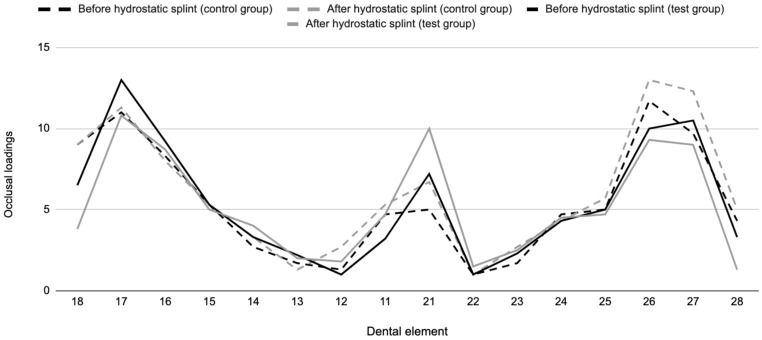
Occlusogram: median occlusal loadings on dental elements in the non-TMDs and TMDs groups (expressed as percentages).

To obtain a full view of the occlusal force distribution pattern in the two groups before and after using the hydrostatic splint, the recorded data for single teeth were added to a Cartesian axis system, producing a graph defined as an occlusogram for each group (see Figure 1).

### 3.3. Percentage Force Distribution per Sector

The percentage force distribution in the anterior and posterior regions of the non-TMDs and TMDs groups is shown in Table 5. The analysis of force distributions in the anterior and posterior right regions before and after hydrostatic splint therapy showed significant differences within the population (*p* < 0.001; *p* = 0.021, respectively), highlighting changes in specific areas of the mouth. No significant differences were found in the non-TMDs group between the anterior (*p* = 0.074), posterior left and right percentages forces (*p* = 0.993 and *p* = 0.597) before and after using the hydrostatic splint. However, in the Wilcoxon signed-rank test, a significant difference was found for anterior (*p* = 0.001) and posterior right (*p* = 0.022) percentages of forces before and after using the hydrostatic splint in the TMDs group. Although, the forces were 32–56% higher in the front region (tooth No. 13–23) after the hydrostatic splint wearing. Contrary to the anterior region, occlusal force in the posterior area was reduced after using the hydrostatic splint compared to natural maximum intercuspation before. This information indicates a new occlusal distribution outcome after using the hydrostatic splint.

The analysis of teeth contributing to occlusion at maximum intercuspation before and after the hydrostatic splint showed a statistically significant difference among the groups of the study (*p* = 0.002).

The maximum percentage occlusal force detected on any tooth in the non-TMDs and TMDs groups was reduced after using the hydrostatic splint compared to natural maximum intercuspation before, accordingly 27.7, 26.0 and 39.0, 35.0. This parameter once again demonstrates an occlusal force redistribution outcome after using the hydrostatic splint (see Table 5).

### 3.4. Percentage Force Distribution per Side

Assessment of the occlusal forces on the right and left sides was performed considering the median in two groups and all of the population. Obtained values are shown in Table 5. In the Wilcoxon signed-rank test, no significant difference was found in the non-TMDs and TMDs groups before and after using the hydrostatic splint. In addition, there were no significant differences in the population before and after using the hydrostatic splint. However, the percentage occlusal force distribution between the left and right sides was more equivalent after using the hydrostatic splint (non-TMDs group: left side 50.0%, right side 50.0%; TMDs group: left side 50.4%, right side 49.6%). Specifically, the results show that the occlusal force distribution between the left and right sides was more equivalent after using the splint, as demonstrated by the statistical analysis presented in Table 5.

## 4. Discussion

The findings of this study support the hypothesis that the use of the hydrostatic splint improves the distribution of occlusal forces in both non-TMD and TMD groups, leading to a more balanced occlusion. Specifically, significant changes were observed in the anterior and posterior right regions of the TMD group, indicating that the hydrostatic splint effectively redistributes occlusal forces, thereby contributing to improved occlusal balance. This study implies that almost all patients had an unbalanced bite, which the hydrostatic splint was correcting. The results confirm that hydrostatic splint therapy can be a valuable tool in managing occlusal imbalances, particularly in patients with TMD, and underscores its potential as a preparatory step for full-mouth rehabilitation.

Our findings align with those of Ferrato et al. [3], who also found differences in occlusal force distribution between healthy subjects and TMDs patients. The researcher detected that the area with the highest load was located at the mesiopalatal cusp of the first upper molars, whereas Ma et al. [10] claimed that maximum occlusal loading was located in an area of the second molar. Similar findings were detected in the present study: maximum occlusal loading was detected in the area of the first upper molar in the non-TMDs group, and it was detected in the second upper molar in the TMDs group.

Specifically, T-Scan analysis of the non-TMDs and TMDs groups revealed that use of the hydrostatic splint was associated with a decrease in occlusal load in the posterior region and an increase in the frontal area. In the non-TMDs group, this change was less pronounced, with patients tending to maintain their preexisting occlusion. In contrast, the TMDs group displayed a greater tendency toward adopting a new occlusion, characterized by stronger occlusal load in the frontal region and weaker occlusal load in the posterior region. This shift in occlusal balance was accompanied by movement of the mandible in a caudoventral direction, which relieved pressure on the bilaminar zone of the discus by putting the condyles in a more central position of mandibular fossa and improving the neuromuscular balance, as demonstrated by Buchbender et al. [14]. These findings prove that the hydrostatic splint is effective even after a relatively short period of wear.

The percentage of the occlusal forces on the right and left sides was not statistically significant between the two groups in the study sample. However, within the TMDs group, the difference in occlusal force between the right and left sides was higher (before splint: left side 49.2%, right side 50.9% and after splint: left side 50.4%, right side 49.6%; Table 5) compared to the non-TMDs group (before splint: left side 50.2%, right side 49.8% and after splint: left side 50.0%, right side 50.0%; Table 5), indicating greater asymmetry in occlusal forces among TMD patients. However, the results of the present study showed that the hydrostatic splint significantly improved occlusal symmetry: the occlusal force distribution between the left and right sides was more equivalent after using the hydrostatic splint (non-TMDs group: left side 50.0%, right side 50.0%; TMDs group: left side 50.4%, right side 49.6%; Table 5). The results in the study by Dzingute et al. [27] were similar: the average of the asymmetry index of the occlusal force (the difference of the occlusal force between the right and the left sides) was 15.90, SD = 12.10 in the group of patients who complained of TMDs and 12.93, SD = 9.19 in healthy subjects. Wang et al. [28] also detected similar results and the greater bilateral asymmetry in the occlusal force: the average of the asymmetry index of maximum occlusal force was 16.66, SD = 0.47 in patients who exhibited TMDs. Occlusal forces between the left and right sides of the mandible are distributed unevenly in patients with TMDs, which results in the activity of masticatory muscles on one or two sides and may determine TMDs [27]. However, the results of the present study showed that the hydrostatic splint significantly improved occlusal symmetry: the occlusal force distribution between the left and right sides was more equivalent after using the hydrostatic splint. Gözler et al. [29] came to the same conclusion: values of bite forces on both sides of the mandible were more balanced after the splint therapy; that is, before and after usage of the splint, values were 45.26% and 47.87% on the left side and 57.24% and 54.63% on the right side, respectively. These findings suggest that the hydrostatic splint may be effective in improving occlusal force distribution in all adult patients, both healthy and those with TMDs. Moreover, research by Ferrato et al. [3] corroborated these findings, noting that hydrostatic splints reduced occlusal load discrepancies and enhanced muscle function symmetry in TMD patients.

A few limitations in this study need to be clearly listed and critically discussed. First, the effect of hydrostatic splint therapy was not investigated over a longer follow-up period; the study only assessed the primary effects after the first 30 min of using the hydrostatic splint. Second, all subjects received the same hydrostatic splint model with a medium volume, which may not have been ideal for all participants. Some subjects might have benefited from a smaller or larger volume splint depending on their tooth wear. Third, the different TMJ anatomies (e.g., ligaments and articular disc variations) among subjects were not considered, which could have influenced the results. Fourth, the study did not evaluate masticatory and neck muscles, which could be meaningful for future clinical studies. Evaluating muscle tension could provide insights into the time needed to achieve the primary effects of hydrostatic splint therapy.

It is important to note that the study sample was relatively small and conducted at a single dental practice, which may limit the generalizability of the findings. Future research with larger sample sizes and multiple study sites would be beneficial in further exploring the effects of the hydrostatic splint on patients with TMD symptoms and occlusal forces. The use of validated assessment tools and longer-term follow-up would provide more reliable data on the effectiveness of the hydrostatic splint in managing central relations. Additionally, it would be interesting to investigate the optimal treatment protocol for using the hydrostatic splint in patients with TMD, including the frequency and duration of treatment.

## 5. Conclusions

Hydrostatic splint therapy significantly improves occlusal force distribution, leading to a more balanced occlusion in both non-TMD and TMD patients. The therapy effectively reduces occlusal loads in the posterior regions while increasing them in the anterior regions, especially in TMD patients. These findings support the use of hydrostatic splint therapy as a valuable component of full-mouth rehabilitation. For patients requiring full-mouth rehabilitation, hydrostatic splint therapy helps establish a balanced occlusal foundation, which is essential for the success of subsequent restorative treatments. Additionally, the digitized occlusion examination method used in this study proved to be reliable and repeatable, underscoring the clinical utility of hydrostatic splint therapy in dental practice.

## Figures and Tables

**Table 1:** General characteristics of the patients.

	Non-TMDs Group	TMDs Group	All Participants	*p* Value
Number (n)	19	32	51	
* Age (years)	25.4, SD = 4.8 (20–38)	27.4, SD = 7.0 (22–53)	26.7, SD = 6.3 (20–53)	0.208
Gender n (%)				0.473
Female	15 (78.9)	25 (78.1)	40 (78.4)	
Male	4 (21.1)	7 (21.9)	11 (21.6)	

* Data are presented as mean, SD (range).

**Table 2:** Frequencies of replies to the four questions of the BRUX scale (n = 51).

	Never (%)	Sometimes (%)	Regularly (%)	Often (%)	Always (%)
Sleep clenching	21.6	23.5	21.6	23.5	9.8
Sleep grinding	52.9	9.8	17.6	15.7	3.9
Awake clenching	7.8	25.5	43.1	23.5	0.0
Awake grinding	80.4	11.8	5.9	2.0	0.0

**Table 3:** Characterization of all participants according to age, gender, and TMDs severity degrees (n = 51).

	Non-TMDs Group	Mild TMDs	Moderate TMDs	Severe TMDs	*p* Value
Sample n (%)	19 (37.3%)	23 (45.1%)	8 (15.7%)	1 (2.0%)	
* Age (years)	25.4, SD = 4.8 (20–38)	28.5, SD = 8.0 (22–53)	24.0, SD = 1.1 (23–26)	28 (28–28)	0.181
Gender n (%)					
Female	15 (78.9)	18 (78.3)	6 (75.0)	1 (100.0)	0.953
Male	4 (21.1)	5 (21.7)	2 (25.0)	0 (0.0)	

* Data are presented as mean, SD (range).

**Table 4:** Values of occlusal loadings on dental elements are presented as median [IQR, 25th–75th percentile] in the non-TMDs and TMDs group (expressed as a percentage).

Dental Element	17	16	15	14	13	12	11	21	22	23	24	25	26	27
Before splint (non-TMDs)	11.0 [7.3–15.7]	8.3 [6.0–10.7]	5.3 [3.0–7.0]	2.7 [2.0–5.3]	1.7 [1.0–2.3]	1.3 [0.3–4.0]	4.7 [1.3–10.7]	5.0 [2.0–11.0]	1.0 [0.3–3.3]	1.7 [1.9–3.3]	4.7 [2.7–7.7]	5.0 [3.0–7.7]	11.7 [7.3–16.3]	9.7 [7.7–14.3]
After splint (non-TMDs)	11.3 [6.0–13.7]	8.0 [6.7–12.00]	5.3 [4.0–6.7]	3.3 [2.0–5.3]	1.3 [0.7–2.0]	2.7 [0.3–3.3]	5.3 [1.7–10.3]	6.7 [3.0–11.3]	1.0 [0.7–2.7]	2.7 [1.7–4.0]	4.3 [2.0–7.0]	5.7 [3.3–9.0]	13.0 [7.3–15.7]	12.3 [5.7–15.0]
*p*-Value (non-TMDs)	0.126	0.711	0.406	0.059	0.938	0.342	0.224	0.102	0.860	0.239	0.962	0.017	0.372	0.222
Before splint (TMDs)	13.0 [10.0–15.8]	9.2 [6.4–13.6]	5.3 [3.1–6.7]	3.3 [2.1–6.3]	2.2 [1.0–3.9]	1.0 [0.4–2.9]	3.2 [2.0–7.3]	7.2 [1.8–12.9]	1.0 [0.4–2.6]	2.3 [0.8–3.9]	4.3 [2.1–5.7]	5.0 [3.1–6.3]	10.0 [7.3–13.8]	10.5 [6.3–15.7]
After splint (TMDs)	10.8 [6.8–13.8]	8.7 [5.8–13.3]	5.0 [2.7–6.7]	4.0 [2.1–7.3]	2.0 [1.3–3.7]	1.8 [0.7–5.6]	4.7 [3.1–8.3]	10.0 [4.1–15.5]	1.5 [0.7–2.9]	2.5 [0.8–5.4]	4.5 [1.8–6.3]	4.7 [3.0–7.0]	9.3 [6.3–12.3]	9.0 [5.1–14.7]
*p*-Value (TMDs)	0.016	0.793	0.797	0.071	0.493	0.014	0.008	0.003	0.546	0.097	0.904	0.567	0.135	0.79

**Table 5:** T-Scan analysis parameters per sector and per side of the non-TMDs and TMDs groups before and after using the hydrostatic splint expressed as percentage.

Median [IQR, 25th–75th Percentile]	Non-TMDs Group			TMDs Group		
	Before Hydrostatic Splint	After Hydrostatic Splint	*p* Value	Before Hydrostatic Splint	After Hydrostatic Splint	*p* Value
Anterior region	19.0 [10.0–31.0]	25.0 [13.3–32.3]	0.074	18.3 [9.8–35.9]	28.5 [15.4–42.3]	0.003
Posterior right	39.0 [34.3–49.0]	37.7 [32.3–47.0]	0.673	41.3 [33.3–47.3]	39.2 [24.8–43.3]	0.022
Posterior left	38.7 [31.0–48.7]	36.7 [30.7–50.0]	1.21	37.8 [24.2–44.3]	32.2 [22.8–40.8]	0.126
Teeth in occlusion	14.3 [13.3–15.3]	15.0 [14.7–15.7]	0.184	14.2 [12.8–15.0]	14.2 [12.3–14.7]	0.576
Left side	50.2 [43.8–55.1]	50.0 [43.4–52.6]	0.747	49.2 [42.9–55.4]	50.4 [44.1–55.9]	0.315
Right side	49.8 [44.9–56.2]	50.0 [47.4–56.6]	0.747	50.9 [44.7–57.1]	49.6 [44.1–55.9]	0.315

## Data Availability

The original contributions presented in the study are included in the article, further inquiries can be directed to the corresponding author.
